# Added Sugar, Macro- and Micronutrient Intakes and Anthropometry of Children in a Developing World Context

**DOI:** 10.1371/journal.pone.0142059

**Published:** 2015-11-11

**Authors:** Eleni M. W. Maunder, Johanna H. Nel, Nelia P. Steyn, H. Salome Kruger, Demetre Labadarios

**Affiliations:** 1 School of Agricultural, Earth and Environmental Sciences, University of KwaZulu-Natal, KwaZulu-Natal, South Africa; 2 Department of Logistics, University of Stellenbosch, Stellenbosch, Western Cape, South Africa; 3 Division of Human Nutrition, University of Cape Town, Cape Town, South Africa; 4 North West University, Potchefstroom; North West Province, South Africa; 5 Population Health, Health Systems and Innovation, Human Sciences Research Council, Cape Town, Western Cape, South Africa; Medical University Innsbruck, AUSTRIA

## Abstract

**Objective:**

The objective of this study was to determine the relationship between added sugar and dietary diversity, micronutrient intakes and anthropometric status in a nationally representative study of children, 1–8.9 years of age in South Africa.

**Methods:**

Secondary analysis of a national survey of children (weighted n = 2,200; non weighted n = 2818) was undertaken. Validated 24-hour recalls of children were collected from mothers/caregivers and stratified into quartiles of percentage energy from added sugar (% EAS). A dietary diversity score (DDS) using 9 food groups, a food variety score (FVS) of individual food items, and a mean adequacy ratio (MAR) based on 11 micronutrients were calculated. The prevalence of stunting and overweight/obesity was also determined.

**Results:**

Added sugar intake varied from 7.5–10.3% of energy intake for rural and urban areas, respectively. Mean added sugar intake ranged from 1.0% of energy intake in Quartile 1 (1–3 years) (Q1) to 19.3% in Q4 (4–8 years). Main sources of added sugar were white sugar (60.1%), cool drinks (squash type) (10.4%) and carbonated cool drinks (6.0%). Added sugar intake, correlated positively with most micronutrient intakes, DDS, FVS, and MAR. Significant negative partial correlations, adjusted for energy intake, were found between added sugar intake and intakes of protein, fibre, thiamin, pantothenic acid, biotin, vitamin E, calcium (1–3 years), phosphorus, iron (4–8 years), magnesium and zinc. The prevalence of overweight/obesity was higher in children aged 4–8 years in Q4 of %EAS than in other quartiles [mean (95%CI) % prevalence overweight 23.0 (16.2–29.8)% in Q4 compared to 13.0 (8.7–17.3)% in Q1, p = 0.0063].

**Conclusion:**

Although DDS, FVS, MAR and micronutrient intakes were positively correlated with added sugar intakes, overall negative associations between micronutrients and added sugar intakes, adjusted for dietary energy, indicate micronutrient dilution. Overweight/obesity was increased with higher added sugar intakes in the 4–8 year old children.

## Introduction

Increasing intakes of sugar in the diet have been of concern to World Health Organization (WHO) [1, 2] in relation to obesity, non-communicable diseases, dental caries, food patterns and nutrient intakes. Substantial evidence exists for the relationship of a high sugar intake and dental caries [3–6]. The effect of added sugar intake on micronutrient dilution has been less consistent [7–11]. In relation to the latter relationship a number of reviews [8, 9, 11–15] on the effects of sugar intake on micronutrients intake, are primarily based on studies of adults and children from developed countries, with the exception of one small study in the elderly in South Africa [16]. The need for further studies in developing countries has been highlighted [14]. Since 2007, a study on sugar and micronutrient intakes in adults in South Africa has been published [17]. An analysis of sugar and micronutrient intakes in children aged 6–9 years in South Africa at the national level supported the inclusion of a Food Based Dietary Guideline on sugar consumption [18]. Since food patterns and the dietary context in children from developed countries are very different to those in developing countries, where a large proportion of children generally do not meet the recommended nutrient requirements, extrapolation of findings from the developed to the developing countries is both limiting and inappropriate. Further, the findings in the developed countries often lack context in terms of consequences of the relationship with regard to growth and nutritional status.

Many children in developing countries suffer the effects of food insecurity resulting in deficits in energy, protein, and micronutrients such as vitamin A, iron, and zinc. These deficits are associated with low weight- and low height- for- age as well as micronutrient deficiencies [19–21]. Generally the diet of these children is monotonous with low dietary diversity scores and comprises large portions of carbohydrates (staple foods) and lower intakes of animal proteins, fruit and vegetables [22]. More specifically, in South Africa a large proportion of the population live below the poverty level [23] and underweight affects one in ten children; stunting one in five children; and vitamin A, iron and zinc deficiencies are common [24, 25]. Tea with added sugar is very commonly consumed, frequently more than once a day by both adults and children [26]. Table sugar is fairly inexpensive and used by more than 97% of households [26]. Because of a lack of knowledge of differences in food groups consumed and the possible effects of micronutrient dilution with increased levels of added sugar, research in this field is essential, since micronutrient intakes of children are already compromised by poor intakes in many households.

In the context of energy intake, it is also important that the rising trends in overweight and obesity in children [2] are considered. The 2012 South African National Health and Nutrition Examination Survey (SANHANES-1) showed a combined overweight and obesity prevalence of 13.5% for South African children aged 6–14 years [27]. The report concluded that, “interventions are needed to address the dual problems of chronic undernutrition (stunting) and the rapidly rising trend of overweight and obesity among children in South Africa”.

Sugar in the diet, especially in the form of sugar sweetened beverages (SSB) has been implicated in the development of obesity [28]. Further evidence comes from a systematic review and meta-analysis which combined the findings from 38 cohort studies and 30 randomized, controlled trials (Te Morenga, Mallard & Mann, 2012) [29]. Findings from the studies on adults with ad libitum diets showed that the effects of sugar intake on weight were due not only to SSBs but also to the total intake of sugar. An increased sugar intake was associated with a 0.75 kg higher body weight while a decreased sugar intake was associated with a 0.80 kg lower body weight. Most of the studies on children in the review [29] investigated SSB with few investigating total/added sugar intake. The link of SSB intake in children with increase in weight or adiposity is supported by several reviews of prospective studies in school-aged children [29–31]. The odds ratio for children being overweight or obese was 1.55 (1.32–1.82) among groups with the highest intake of SSB, compared to the lowest intake [29]. One daily serving increase in SSB for children was associated with a 0.06 (0.02–0.10) increase in BMI [30]. However with regard to the five studies reviewed which investigated total/added sugar in children [29] none of them found a difference in overweight measures between higher and lower consumers of sugar [32–36]. Studies investigating the relationship between socioeconomic status and sugar intakes have shown that in developed countries sugar sweetened beverage/soda consumption is higher in children in low income households [37–39] and in children whose mothers were less educated [40]. However the opposite association was seen in a study in Brazil where greater food insecurity was inversely associated with sugar intake [41].

The objective of this secondary analysis was therefore to determine the relationship between added sugar and food group intakes, dietary diversity, micronutrient intakes and anthropometric status in a nationally representative study of children in a developing country. This objective was achieved.

## Methods

### Ethics

This research was a secondary data analysis and no ethical approval was required. The original study was approved by the Ethics Committee of the University of Stellenbosch, South Africa.

### Sample

A secondary data analysis was undertaken on the database of the National Food Consumption Survey (NFCS) which took place in 1999 [24, 42]. To date the 1999 NFCS is the only national survey which has measured dietary intake by 24 hour recall and food frequency questionnaire. Data was collected on children ages 1.0–8.9 years old in a national sample. The original sample was oversampled for poorer areas. For this analysis the data was weighted for provincial, urban/rural and age representation (weighted n = 2,200, non weighted n = 2818). The sampling procedures have been described elsewhere [43].

### Dietary intake

A 24 hour recall interview using a pre-coded, validated questionnaire [24, 43] was conducted with the mother or caregiver of each of the children participating in the study at their homes. Trained interviewers conducted the dietary recalls which required each participant to recall all food and beverages consumed in the previous 24 hours. Dietary aids comprising food models of local foods and portion sizes were used to assist the interviewers in obtaining dietary information. Relative validity was determined by comparison with data obtained from the same participants using a food-frequency questionnaire and three 24-hour recalls were repeated on 10% of the sample for quality control purposes. The dietary intake results have been reported elsewhere [26]. Signed informed consent was obtained from the caregivers of the children who were participants in the study.

Dietary diversity was measured by means of a dietary diversity score (DDS) which was calculated out of a maximum of 9 food groups, namely (1) cereals, roots and tubers; (2) vitamin-A-rich fruits and vegetables; (3) other fruit; (4) other vegetables; (5) legumes and nuts; (6) meat, poultry and fish; (7) fats and oils; (8) dairy; and (9) eggs [44]. These food groups were also used to examine food consumption of each food group. A food variety score (FVS) was calculated as the total number of different food items consumed over a period of 24 hours [44]. The nutrient adequacy ratio (NAR) was calculated for micronutrients as the intake of the nutrient divided by the recommended nutrient intake (RNI) for a given nutrient expressed as a percentage. Micronutrients included vitamins A, B_6_, B_12_ and C, thiamin, riboflavin, niacin, folate, calcium, iron and zinc. Mean adequacy ratio (MAR) was calculated as the sum of the NARs (truncated at 100%) divided by the number of nutrients (= 11). (Truncation before calculating MAR was necessary to reduce all those NARs above 100% to 100%). Only data for MAR is presented.

### Anthropometric data

Weight was measured to the nearest 100g by trained fieldworkers, average of two measurements, using electronic scales, placed on an even, uncarpeted area and levelled with the aid of its in-built spirit level. The children were weighed (preferably after emptying their bladders) and with the minimum of clothing. Diapers only for babies (dry only) or underclothes for older children were allowed. Supine length, for children younger than 2 years, was determined by means of a measuring board. Two readings were taken (average reported) and the measurement was repeated if the two readings varied by more than 0.5 cm. For children 2 years of age and older, standing height was taken by means of a stadiometer [24, 45]. Height-for-age, weight-for-age and body mass index (BMI)-for-age Z-scores were calculated using the WHO Anthro (1–5 years) and WHO AnthroPlus (5–9 years) software. Height-for-age Z-score <-2 was defined as stunting[46, 47] and BMI-for-age Z-scores of >+1 to +2 and >+2 were defined as overweight and obese respectively in children aged 5.1 years and older [46]; BMI-for-age Z-of >+2 to +3 and >+3 were defined as overweight and obese respectively in children aged 1–5 years old [47].

### Data analysis

Data was analysed in SAS 9.3 (SAS Institute Inc., SAS Version 9.3 Cary, NC, USA). Mean values with 95% confidence intervals were calculated for energy, carbohydrate and added sugar intakes in 1–3 and 4–8 year olds. Data was also analysed by rural and urban areas, and by categories of money spent by household on food weekly as a measure of socio-economic status (SES). Added sugar was defined as sugar (sucrose) added at household level and sugars (mono- or disaccharides) added at the point of manufacture (ingredients in food processing). The amount of added sugar was determined by the nutrient tag ‘SUGAR’ in the food composition database. White and brown sugar (sucrose), as well as honey and syrup were counted as added sugar by the food composition database compilers. Sugar added at the table refers to white and brown sugar added to food or drinks, combined together (this relates to food code 3989 and food code 4005 respectively in the national food composition database) [48]. The added sugar value does not include sugars naturally present in foods, e.g. lactose in milk, fructose, glucose and sucrose in fruit or fruit juice. Free sugars include monosaccharides and disaccharides added to foods and beverages by the manufacturer, cook or consumer, and sugars naturally present in honey, syrups, fruit juices and fruit juice concentrates as defined by WHO [2]. Free sugars were calculated by using the values of total carbohydrate in fruit juice and fruit concentrates.

The subjects were divided into quartiles of added sugar intake (% of dietary energy, %EAS). Mean micronutrient intake values were calculated per quartile of sugar intake (%EAS). The South African food composition tables were used [48]. Mean DDS, FVS and MAR were calculated per quartile of % EAS. Pearson’s correlations were determined between sugar intake and micronutrient intakes and micronutrient intake per 4.18MJ, respectively. Pearson’s correlations adjusted for energy intake were also determined between added sugar intake (absolute) and micronutrient intakes. The Pearson partial correlation was used to measure the strength of the linear relationship between nutrient intakes and (absolute) added sugar intake, while adjusting for the effect of energy intake. Food group consumption and anthropometric status (height for age, weight for age, BMI for age and prevalence of overweight/obesity) were also analysed in quartiles of % EAS.

## Results

At the national level, there were significant urban rural differences in daily energy intake, as well as macronutrient intake and related energy distribution indices (Table 1). Children from rural areas had lower mean such values than urban children, except for carbohydrate intake, for which similar or increased intakes were found between the two groups. Mean added sugar intake, as well as % EAS was significantly higher in urban areas (32.4g and 10.3%, respectively) when compared with rural areas (20.9g and 7.5%, respectively).

**Table 1:** Sugar, macronutrient intakes and % contribution of main sources of sugar for children aged 1–8 years by geotype.

Nutrient	1–8 years			1–3 years		4–8 years	
	SA	SA Urban	SA Rural	SA Urban	SA Rural	SA Urban	SA Rural
***Number (weighted n)***	*2818 (2200)*	*1390 (1218)*	*1428 (982)*	*664 (445)*	*644 (350)*	*726 (773)*	*784 (632)*
**Energy (kJ) Mean**	5047.7	5220.0	4834.0^###^	4388.8	4092.4^&&^	5699.3	5244.5^$$^
**Energy (kJ) 95% CI** *	4928–5166	5036–5404	4697–4971	4193–4585	3923–4261	5477–5922	5049–5440
**Protein (g) Mean**	37.3	39.9	34.0^###^	32.7	28.9^&&&^	44.0	36.8^$$$^
**Protein (g) 95% CI**	36.2–38.4	38.3–41.5	32.6–35.5	31.0–34.4	27.6–30.3	42.1–45.9	34.9–38.8
**Fat (g) Mean**	30.0	35.3	23.3^###^	29.0	21.5^&&&^	39.0	24.4^$$$^
**Fat (g) 95% CI**	28.7–31.2	33.2–37.4	22.1–24.6	27.0–31.0	20.1–22.9	36.3–41.6	22.7–26.0
**Carbohydrate (g) Mean**	183.0	179.1	187.9^##^	153.4	156.2	193.9	205.5^$$^
**Carbohydrate (g) 95% CI**	179.1–186.9	173.4–184.8	182.9–192.9	146.6–160.3	149.5–162.9	187.2–200.7	198.3–212.7
**AS (g) Mean**	27.3	32.4	20.9^###^	25.4	17.3^&&&^	36.5	22.9^$$$^
**AS (g) 95% CI**	25.7–28.9	29.7–35.2	19.3–22.5	23.3–27.5	15.9–18.6	33.0–40.0	20.6–25.3
**Protein (%E) Mean**	12.3	12.7	11.8^###^	12.4	11.7^&&^	12.9	11.8^$$$^
**Protein (%E) 95% CI**	12.1–12.5	12.5–13.0	11.5–12.0	12.0–12.7	11.4–12.1	12.7–13.2	11.4–12.1
**Fat (%E) Mean**	21.2	24.1	17.7^###^	23.7	19.4^&&&^	24.3	16.8^$$$^
**Fat (%E) 95% CI**	20.7–21.8	23.3–24.9	17.0–18.4	22.6–24.7	18.4–20.3	23.4–25.3	16.0–17.5
**CHO (%E) Mean**	65.8	62.4	70.1^###^	63.2	68.5^&&&^	62.0	70.9^$$$^
**CHO (%E) 95% CI**	65.3–66.4	61.6–63.3	69.3–70.8	62.1–64.4	67.3–69.6	61.0–63.0	70.1–71.8
**AS (%E) Mean**	9.1	10.3	7.5^###^	9.8	7.5^&&&^	10.6	7.6^$$$^
**AS (%E) 95% CI**	8.7–9.5	9.7–11.0	7.1–8.0	9.1–10.5	6.9–8.2	9.8–11.4	6.9–8.2
**% Consuming white sugar**	77.0	80.3	72.8	77.9	74.2	81.7	72.1
**White sugar % of Added Sugar intake**	60.1	51.0	77.7	56.2	82.8	48.9	75.5
**% Consuming Cool Drink Squash Type**	14.8	20.7	7.5	16.5	3.6	23.2	9.6
**Cool Drink Squash Type % AS intake**	10.4	12.7	6.1	10.6	3.1	13.5	7.3
**% Consuming Cool Drink Carbonated**	5.3	8.2	1.6	4.8	1.8	10.2	1.6
**Cool Drink Carbonated % AS intake**	6.0	8.2	1.7	4.1	2.3	9.9	1.4
**WS+CDC+CDS % AS intake**	76.5	71.9	85.5	70.9	88.2	72.3	84.2

* 95% CI = 95% Confidence Intervals: LCI = Lower confidence interval; UCI = Upper confidence interval

^###^Significant difference between urban and rural values, t-test p<0.0001

^##^Significant difference between urban and rural values, t-test p<0.01

^&&&^Significant difference between urban and rural values, age 1–3 years, t-test p<0.0001

^&&^Significant difference between urban and rural values, age 1–3 years, t-test p<0.01

^$$$^Significant difference between urban and rural values, age 4–8 years, t-test p<0.0001

^$$^Significant difference between urban and rural values, age 4–8 years, t-test p<0.01

CHO = carbohydrate; WS = White sugar; AS = Added sugar; CDC = Cool drink, carbonated; CDS = Cool drink, squash type

The top 3 sources of added sugar in the diet for the country as a whole in terms of contribution (%) to added sugar intake in children aged 1–8 years were white sugar (60.1%), cool drink (squash type; 10.4%), carbonated cool drink (6.0%) (Table 1). The food item consumption rates (%) were 77.0% for white sugar, 14.8% for cool drink (squash type) and 5.3% for carbonated cool drink. Corresponding rates for the next seven top food sources of added sugar (jam/marmalade, hard boiled/soft jelly sweets, cookies/pancakes/cakes/tarts, brown sugar, chocolate/sweets, canned/bottled orange juice, pumpkin/butternut) were less than 10% for consumption rates and less than 5% for contributions to added sugar intakes. These 10 items provided 91.6% of the added sugar in the diet (89.6% in urban and 96.6% in rural areas). Most children (77%) consumed sugar (white and brown) added at the table at breakfast, with only a few (3–9%) adding sugar at other meals. By age and geotype these three food items i.e. white sugar, carbonated cool drinks and squash type cool drinks (Table 1) comprised 70.9% (urban) - 88.2% (rural) of the added sugar intake for 1–3 year old children. The consumption rates and contribution to added sugar intake of cool drinks were higher in older children and in children living in urban areas (Table 1). Added sugar consumption and %EAS increased with increased weekly household food expenditure, as did the consumption of energy, protein and fat, for both age groups (Tables 2 and 3). With increased household food expenditure white sugar comprised a lower proportion of added sugar intake and the contribution of cool drinks (squash type) and carbonated cool drinks increased.

**Table 2:** Sugar, macronutrient intakes and % contribution of main sources of sugar for children aged 1–3 by categories of money spent by household on food weekly (SES).

Nutrient	1–3 years				
	SA	SES 1	SES 2	SES 3	SES 4
***Number (weighted n)***	1308 (795)	317 (194)	244 (149)	228 (141)	228 (140)
**Energy (kJ) Mean**	4258.4	4127.4 [B]	4349.6 [A][B]	4212.2 [A][B]	4656.7 [A]
**Energy (kJ) 95% CI** *	4128.0–4388.9	3889.7–4365.2	4049.3–4649.9	3956.6–4467.8	4366.6–4946.7
**Protein (g) Mean**	31.0	29.1 [B]	31.5 [A][B]	30.7 [B]	35.1 [A]
**Protein (g) 95% CI**	29.9–32.1	27.0–31.2	29.2–33.9	28.4–33.1	32.5–37.6
**Fat (g) Mean**	25.7	21.4 [C]	27.0 [A][B]	26.9 [B]	31.4 [A]
**Fat (g) 95% CI**	24.4–26.9	19.6–23.2	24.0–30.1	24.0–29.9	28.6–34.3
**Carbohydrate (g) Mean**	154.6	158.3	156.7	149.4	161.1
**Carbohydrate (g) 95% CI**	149.8–159.5	148.6–168.0	146.1–167.2	140.3–158.5	151.2–171.0
**Added Sugar (g) Mean**	21.8	18.6 [B]	19.5 [B]	25.5 [A]	29.7 [A]
**Added Sugar (g) 95% CI**	20.5–23.1	16.2–21.0	17.2–21.9	22.4–28.5	26.1–33.3
**Protein (%E) Mean**	12.1	11.7	12.3	12.2	12.5
**Protein (%E) 95% CI**	11.8–12.3	11.2–12.2	11.7–12.8	11.6–12.8	12.0–12.9
**Fat (%E) Mean**	21.8	19.3 [B]	22.5 [A]	23.0 [A]	24.3 [A]
**Fat (%E) 95% CI**	21.1–22.5	18.0–20.5	21.0–24.0	21.6–24.4	22.7–26.0
**Carbohydrate (%E) Mean**	65.5	68.6	65.0	63.9	62.5
**Carbohydrate (%E) 95% CI**	64.7–66.4	67.2–69.9	63.5–66.6	62.3–65.6	60.6–64.3
**Added Sugar (%E) Mean**	8.8	7.9 [B]	7.8 [B]	10.3 [A]	10.6 [A]
**Added Sugar (%E) 95% CI**	8.3–9.3	6.8–9.0	6.8–8.8	9.2–11.5	9.6–11.6
**% Consuming white sugar**	76.2	74.5	72.8	78.3	80.0
**White sugar % of Added Sugar intake**	65.4	74.2	67.6	62.1	50.6
**% Consuming Cool Drink Squash Type**	10.8	5.2	10.5	15.7	19.7
**Cool Drink Squash Type % AS intake**	8.0	4.6	7.7	9.8	11.5
**% Consuming Cool Drink Carbonated**	3.5	2.9	1.4	4.7	7.5
**Cool Drink Carbonated % AS intake**	3.5	4.0	1.5	3.6	5.6
**WS+CDC+CDS % AS intake**	76.9	82.7	76.8	75.5	67.7

* 95% CI = 95% Confidence Intervals: LCI = Lower confidence interval; UCI = Upper confidence interval.

[A], [B],[C],[D]: Significant differences between SES groups when letters are different; Bonferroni, p<0.05.

CHO = carbohydrate; WS = White sugar; AS = Added sugar; CDC = Cool drink, carbonated; CDS = Cool drink, squash type

SES 1 = R0-50; SES 2 = R50-100; SES3 = R100-200; SES4 = R200-400+

**Table 3:** Sugar, macronutrient intakes and % contribution of main sources of sugar for children aged 4–8 years by categories of money spent by household on food weekly (SES).

Nutrient	4–8 years				
	SA	SES 1	SES 2	SES 3	SES 4
***Number (weighted n)***	1510 (1405)	339 (302)	281 (265)	325 (301)	285 (282)
**Energy (kJ) Mean**	5494.6	5309.0 [B]	5275.1 [B]	5496.7 [B]	6206.6 [A]
**Energy (kJ) 95% CI** *	5344.9–5644.4	5021.7–5596.2	4984.2–5566.1	5173.0–5820.5	5872.5–6540.7
**Protein (g) Mean**	40.8	37.6 [B]	39.8 [B]	41.2 [B]	47.1 [A]
**Protein (g) 95% CI**	39.4–42.2	34.7–40.4	37.3–42.4	38.5–43.9	44.2–50.0
**Fat (g) Mean**	32.4	25.4 [C]	31.4 [B]	34.5 [B]	43.5 [A]
**Fat (g) 95% CI**	30.8–34.0	23.0–27.7	28.6–34.1	31.2–37.8	39.6–47.5
**Carbohydrate (g) Mean**	199.1	207.0 [A][B]	190.1 [B]	194.4 [A][B]	209.6 [A]
**Carbohydrate (g) 95% CI**	194.2–204.1	195.5–218.5	179.5–200.6	183.3–205.6	198.7–220.5
**AS (g) Mean**	30.4	23.2 [C]	25.1 [C]	32.3 [B]	45.4 [A]
**AS (g) 95% CI**	28.2–32.6	19.4–27.0	22.2–27.9	28.8–35.9	39.9–50.8
**Protein (%E) Mean**	12.4	11.8 [B]	12.6 [A][B]	12.6 [A][B]	12.7 [A]
**Protein (%E) 95% CI**	12.2–12.6	11.3–12.4	12.2–13.1	12.1–13.1	12.3–13.1
**Fat (%E) Mean**	20.9	17.3 [C]	21.5 [B]	22.3 [B]	25.2 [A]
**Fat (%E) 95% CI**	20.3–21.6	16.2–18.4	20.3–22.7	21.1–23.5	23.7–26.7
**CHO (%E) Mean**	66.0	70.2 [A]	65.5 [B]	64.4 [B]	61.4 [C]
**CHO (%E) 95% CI**	65.3–66.7	69.0–71.4	64.2–66.8	63.1–65.8	59.8–62.9
**AS (%E) Mean**	9.2	7.6 [C]	8.3 [C][B]	9.8 [B]	12.0 [A]
**AS (%E) 95% CI**	8.7–9.8	6.6–8.6	7.4–9.2	8.9–10.8	10.9–13.1
**% Consuming white sugar**	77.4	74.0	75.4	79.7	84.3
**White sugar % of Added Sugar intake**	57.9	76.2	61.8	55.8	42.2
**% Consuming Cool Drink Squash Type**	17.1	11.3	13.7	18.5	31.7
**Cool Drink Squash Type % AS intake**	11.4	8.6	9.6	10.8	16.7
**% Consuming Cool Drink Carbonated**	6.3	2.1	4.3	9.3	13.1
**Cool Drink Carbonated % AS intake**	7.0	2.8	4.6	9.6	11.0
**WS+CDC+CDS % AS intake**	76.3	87.6	76.0	76.2	69.9

* 95% CI = 95% Confidence Intervals: LCI = Lower confidence interval; UCI = Upper confidence interval.

[A], [B],[C],[D]: Significant differences between SES groups when letters are different; Bonferroni, p<0.05.

CHO = carbohydrate; WS = White sugar; AS = Added sugar; CDC = Cool drink, carbonated; CDS = Cool drink, squash type

SES 1 = R0-50; SES 2 = R50-100; SES3 = R100-200; SES4 = R200-400+.

Variation in the intakes of some food groups was observed in the different quartiles of %EAS (Table 4). The per capita mean intakes in grams of the other vegetables (1–3 year olds only), other fruit; fats and oils; meat, poultry and fish; dairy (4–8 year olds only); and eggs (4–8 year olds only) food groups overall increased in the higher quartiles of %EAS intakes. For these food groups, consumer portion sizes showed some tendency to decrease overall with increasing %EAS intakes. However, there were significant increases in the percentage of the group who consumed the particular food group, mostly in both age groups (Table 4).

**Table 4:** Mean dietary intake (24-hr recall) of South African children aged 1–3 and 4–8 years old according to quartiles of added sugar intake as a percentage of energy (%EAS).

	1–3 years				4–8 years			
Sugar quartile	Q1	Q2	Q3	Q4,	Q1,	Q2	Q3	Q4
	1–3 years				4–8 years			
*Number (weighted n)*	*322 (199)*	*317 (199)*	*330 (199)*	*339 (198)*	*387 (351)*	*370 (352)*	*377 (351)*	*376 (351)*
Added Sugar (g) Mean	2.9 [D]	14.1 [C]	25.2 [B]	45.2 [A]	5.8 [D]	20.0 [C]	33.6 [B]	62.1 [A]
Added Sugar (g) 95% CI^**#**^	2.4–3.3	13.3–14.9	23.8–26.5	42.5–47.9	4.9–6.8	19.2–20.8	31.8–35.5	57.5–66.8
Added Sugar (%E) Mean	1.0 [D]	5.4 [C]	9.8 [B]	19.2 [A]	1.5 [D]	6.0 [C]	10.1 [B]	19.3 [A]
Added Sugar (%E) 95% CI	0.8–1.1	5.2–5.5	9.6–10.0	18.2–20.1	1.3–1.8	5.8–6.1	9.9–10.3	18.5–20.1
**Cereals,roots & tubers:**								
Per capita Mean (g)	571	509	473	404	706	631	539	417
Consumers % sample	98	100	100	99	98	100	100	99
Portion size (g) Mean	582 [A]	512 [B]	473 [B]	406 [C]	709 [A]	631 [B]	541 [C]	417 [D]
Portion size (g) 95% CI	541–623	476–547	442–503	378–434	666–751	594–669	510–572	384–449
Portion size (g) Median	500	485	430	360	630	585	515	370
Portion size (g) Q1 –Q3	340–750	314–670	284–615	245–515	400–900	400–785	340–680	245–555
**Vitamin A-rich fruit&veg**:								
Per capita Mean (g)	36	26	21	27	42	23	31	25
Consumers % sample	25	25	19	25	30	18	23	22**
Portion size (g) Mean	144	107	109	106	141	126	135	111
Portion size (g) 95% CI	111–177	91–122	84–133	86–127	122–160	100–151	110–159	90–133
Portion size (g) Median	105	85	85	85	105	90	100	85
Portion size (g) Q1 –Q3	80–190	50–120	45–120	45–125	85–175	80–160	80–160	50–105
**Other fruit**:								
Per capita Mean (g)	30	38	60	64	46	42	61	84
Consumers % sample	13	17	27	30**	15	19	21	34***
Portion size (g) Mean	223	220	221	216	300	219	284	250
Portion size (g) 95% CI	174–271	171–268	192–250	172–261	194–406	190–249	237–331	209–291
Portion size (g) Median	165	150	195	150	200	200	220	180
Portion size (g) Q1 –Q3	125–275	75–250	135–320	75–300	150–270	150–275	150–400	150–330
**Other vegetables**:								
Per capita Mean (g)	19	27	26	23	41	29	35	29
Consumers % sample	20	33	33	29*	33	28	33	34
Portion size (g) Mean	98	83	80	77	126 [A]	102 [A][B]	108 [A][B]	85 [B]
Portion size (g) 95% CI	71–125	72–94	70–89	66–88	103–149	88–117	89–128	72–97
Portion size (g) Median	75	70	70	65	85	85	75	75
Portion size (g) Q1 –Q3	38–115	40–110	40–105	45–90	60–160	55–140	60–120	45–105
**Legumes and nuts:**								
Per capita Mean (g)	18	28	17	13	33	30	20	10
Consumers % sample	15	22	20	16	18	24	20	20
Portion size (g) Mean	118	126	84	79	177 [A]	125 [B]	102 [B][C]	51 [C]
Portion size (g) 95% CI	87–148	95–157	58–111	52–105	134–220	93–156	76–127	36–65
Portion size (g) Median	120	120	50	50	125	85	50	20
Portion size (g) Q1 –Q3	30–135	17–180	10–120	10–120	85–240	12–200	10–170	10–85
**Fats and oils:**								
Per capita Mean (g)	3	3	6	5	5	7	7	9
Consumers % sample	20	31	35	40**	23	44	43	52***
Portion size (g) Mean	13 [A][B]	9 [B]	16 [A]	13 [A][B]	21	17	17	17
Portion size (g) 95% CI	9–16	8–11	11–20	11–15	15–28	13–20	15–19	15–20
Portion size (g) Median	10	7	10	10	14	10	14	14
Portion size (g) Q1 –Q3	5–17	5–10	5–15	5–15	7–21	7–20	7–20	7–21
**Meat, poultry and fish:**								
Per capita Mean (g)	36	48	45	48	56	71	75	68
Consumers % sample	36	50	52	55**	50	58	60	61**
Portion size (g) Mean	100	95	86	88	113	121	125	111
Portion size (g) 95% CI	81–120	83–107	76–96	79–98	101–124	111–132	111–138	98–123
Portion size (g) Median	85	80	72	75	90	105	100	85
Portion size (g) Q1 –Q3	45–110	42–120	40–105	42–105	60–150	60–170	60–166	50–150
**Dairy**:								
Per capita Mean (g)	220	200	163	122	94	102	118	119
Consumers % sample	57	65	67	62	40	52	57	61***
Portion size (g) Mean	386 [A]	306 [B]	241[B][C]	197 [C]	234	197	208	195
Portion size (g) 95% CI	326–446	267–345	207–276	171–223	188–281	166–229	175–240	165–225
Portion size (g) Median	250	250	180	145	165	145	155	135
Portion size (g) Q1 –Q3	125–520	120–430	80–310	60–310	80–305	45–260	68–250	50–273
**Eggs:**								
Per capita Mean (g)	7	10	11	9	6	12	12	10
Consumers % sample	9	14	17	13	8	15	17	14**
Portion size (g) Mean	73	71	68	65	83	81	76	71
Portion size (g) 95% CI	62–84	59–82	61–75	56–74	65–100	70–92	67–85	62–80
Portion size (g) Median	52	52	52	52	55	62	52	52
Portion size (g) Q1 –Q3	52–104	52–100	52–100	52–80	52–104	52–104	52–104	50–102
**White sugar:**								
Per capita Mean (g)	2	11	17	27	4	16	20	30
Consumers % sample	31	88	93	93***	37	92	91	89***
Portion size (g) Mean	8 [D]	12 [C]	19 [B]	28 [A]	11 [C]	17 [B]	22 [B]	34 [A]
Portion size (g) 95% CI	7–8	12–13	18–20	27–30	11–12	17–18	21–24	30–38
Portion size (g) Median	6	12	18	24	12	18	18	24
Portion size (g) Q1 –Q3	6–12	9–12	12–24	18–36	9–12	12–18	12–30	18–36
**Cool drink, squash type:**								
Per capita Mean (g)	1	9	30	70	6	20	70	128
Consumers % sample	1	6	13	24***	2	9	24	33***
Portion size (g) Mean	184	152	228	296	238 [B]	219 [B]	292 [A][B]	390 [A]
Portion size (g) 95% CI	97–272	109–196	201–254	255–338	132–345	191–246	263–322	345–434
Portion size (g) Median	125	125	250	250	250	250	250	330
Portion size (g) Q1 –Q3	125–250	120–250	175–250	180–360	125–250	150–250	250–350	250–500
**Cool drink, carbonated:**								
Per capita Mean (g)	-	0	8	21	0	1	13	69
Consumers % sample		0	4	10***	0	0	6	19***
Portion size (g) Mean	-	125	199	215	125	150	235	366
Portion size (g) 95% CI		125–125	165–233	182–248	125–125	150–150	187–284	303–430
Portion size (g) Median		125	250	180	125	150	250	340
Portion size (g) Q1 –Q3		125–125	125–250	125–250	125–125	150–150	180–250	250–450

^#^95% CI = 95% Confidence interval;

[A], [B], [C], [D]: Bonferroni, p<0.05. Values with different letters are significant differences between groups (%EAS quartile groups)

*Significant relationship between quartile groups of added sugar intake (%EAS) and whether or not food from the relevant food group / food item was consumed, Chi square p<0.05.

**Significant relationship between quartile groups of added sugar intake (%EAS) and whether or not food from the relevant food group / food item was consumed, Chi square p<0.01.

***Significant relationship between quartile groups of added sugar intake (%EAS) and whether or not food from the relevant food group / food item was consumed, Chi square p<0.0001.

In contrast there was a decrease in the per capita consumption of the cereal, roots and tubers in both age groups; vitamin A rich fruits and vegetables (in both age groups), other vegetables (4–8 year olds only); legumes and nuts (4–8 year olds only) and dairy food groups (1–3 year olds only) with increased %EAS intakes. For the vitamin A rich fruits and vegetables food group (4–8 year olds only) there was a significant decrease in the percentage of the group who were consumers. There was a significant decrease in portion size for the cereal, roots and tubers (both age groups); other vegetables (4–8 year olds only); legumes and nuts (4–8 year olds only) and dairy food groups (1–3 year olds only) (Table 4), the reverse being the case for white sugar (both age groups), and cool drink squash type (significant for the 4–8 year olds only). By quartiles of sugar intake (%EAS), both age groups had significantly higher mean values of DDS and FVS in the highest quartile of sugar intake (%EAS) compared with the lowest quartile of sugar intake (%EAS) (Table 5). This was not found for MAR.

**Table 5:** Dietary diversity, food variety score and mean adequacy ratio of 1–3 and 4–8 year olds according to quartiles of added sugar intake per day as a percentage of energy (%EAS).

	All	Q1	Q2	Q3	Q4
**1–3 yrs**					
***Number (weighted n)***	*1308(795)*	*322 (199)*	*317 (199)*	*330 (199)*	*339 (198)*
**Added Sugar (g) Mean**	21.8	2.9 [D]	14.1 [C]	25.2 [B]	45.2 [A]
**Added Sugar (g) 95% CI** *	20.5–23.1	2.4–3.3	13.3–14.9	23.8–26.5	42.5–47.9
**Added Sugar (%) Mean**	8.8	1.0 [D]	5.4 [C]	9.8 [B]	19.2 [A]
**Added Sugar (%) 95% CI**	8.3–9.3	0.8–1.1	5.2–5.5	9.6–10.0	18.2–20.1
**Mean DDS**	3.5	3.0 [B]	3.6 [A]	3.7 [A]	3.7 [A]
**DDS 95% CI**	3.4–3.6	2.8–3.1	3.4–3.8	3.6–3.9	3.5–4.0
**Mean FVS**	5.4	4.1 [B]	5.4 [A]	5.9 [A]	5.9 [A]
**FVS 95% CI**	5.1–5.6	3.9–4.4	5.1–5.8	5.6–6.3	5.5–6.4
**Mean MAR**	64.7	61.4 [B]	68.5 [A]	67.3 [A]	61.7 [B]
**MAR 95% CI**	63.1–66.3	58.2–64.5	66.1–70.9	65.2–69.4	58.4–65.0
**4–8 yrs**					
***Number (weighted n)***	*1510 (1405)*	*387 (351)*	*370 (352)*	*377 (351)*	*376(351)*
**Added Sugar (g) Mean**	30.4	5.8 [D]	20.0 [C]	33.6 [B]	62.1 [A]
**Added Sugar (g) 95% CI**	28.2–32.6	4.9–6.8	19.2–20.8	31.8–35.5	57.5–66.8
**Added Sugar (%) Mean**	9.2	1.5 [D]	6.0 [C]	10.1 [B]	19.3 [A]
**Added Sugar (%) 95% CI**	8.7–9.8	1.3–1.8	5.8–6.1	9.9–10.3	18.5–20.1
**Mean DDS**	3.6	3.2 [C]	3.6 [B]	3.7 [A][B]	4.0 [A]
**DDS 95% CI**	3.5–3.7	3.0–3.3	3.4–3.8	3.5–3.9	3.7–4.2
**Mean FVS**	5.6	4.6 [C]	5.5 [B]	6.0 [A][B]	6.4 [A]
**FVS 95% CI**	5.4–5.9	4.2–4.9	5.2–5.8	5.5–6.4	5.9–6.9
**Mean MAR**	62.5	59.2 [B]	64.9 [A]	64.2 [A]	61.6 [A][B]
**MAR 95% CI**	60.7–64.2	56.7–61.6	62.8–66.9	61.3–67.1	57.4–65.7

*95% CI = 95% Confidence interval;

[A], [B], [C], [D]: Values with different letters are significant differences between groups (%EAS quartile groups), Bonferroni, p<0.05.

DDS = Dietary Diversity Score; FVS = Food Variety Score; MAR = Mean Adequacy Ratio.

A significant positive correlation was shown between added sugar intake per day and absolute micronutrient intakes for all micronutrients except vitamin B_12_ (both age groups) and magnesium (4–8 year olds) (Table 6). However, when nutrient intakes were adjusted for energy intake, significant positive partial correlations were found in 1–3 year olds for % energy carbohydrate, riboflavin, niacin, vitamin B6 and vitamin C (Table 6); and significant negative partial correlations were found between added sugar intake and % energy protein, protein, fibre, thiamin, pantothenic acid, biotin, vitamin E, calcium, phosphorus, magnesium and zinc, indicating that as sugar intake increased, certain nutrient intakes decreased when adjusted for energy intake (Table 6). In 4–8 year olds when nutrient intakes were adjusted for kJ intake significant positive partial correlations were found only between added sugar intake and total fat and riboflavin intakes. Otherwise, partial correlations with added sugar intakes (adjusted for energy intake) were negative and significant for protein (as %E), total protein, fibre, thiamin, pantothenic acid, biotin, vitamin E, phosphorus, iron, magnesium and zinc, indicating that as sugar intake increased, these intakes decreased when adjusted for energy intake. Both the DDS and FVS partial correlations with added sugar intakes were positive and significant indicating that increases in sugar intake were associated with increases in these scores. However, there was no significant partial correlation between added sugar intake and MAR when adjusted for energy intake, indicating no association between overall dietary quality and added sugar intake.

**Table 6:** Pearson correlation coefficients of added sugar intake (AS) per day with nutrient intake values, micronutrient intakes per 4.18MJ, and Pearson’s partial correlation coefficients (adjusted for kilojoule intake).

Variables	Correlation with AS	Correlation with AS / 4.18MJ	Partial correlation with AS†
**Age 1–3 years**			
***Number (weighted n)***	***1308 (795)***	***1308 (795)***	***1308 (795)***
**Carbohydrate (% E)**	-0.01		0.06*
**Protein (% E)**	-0.12***		-0.13***
**Fat (% E)**	0.05		-0.03
**Total carbohydrate**	0.40***		0.04
**Total protein**	0.27***		-0.12***
**Total fat**	0.31***		0.01
**Total fibre**	0.21***		-0.08*
**Vitamin A**	0.06*	-0.06*	-0.01
**Thiamin**	0.19***	-0.24***	-0.23***
**Riboflavin**	0.27***	0.09**	0.09**
**Niacin**	0.31***	0.05	0.12***
**Vitamin B**_**12**_	0.05	-0.01	-0.01
**Vitamin B**_**6**_	0.35***	0.09**	0.13***
**Folic acid**	0.27***	0.07*	0.04
**Pantothenic Acid**	0.20***	-0.12***	-0.16***
**Biotin**	0.14***	-0.14***	-0.10**
**Vitamin D**	0.12***	0.01	0.03
**Vitamin E**	0.09**	-0.09**	-0.14***
**Vitamin C**	0.18***	0.07*	0.10**
**Calcium**	0.08**	-0.11***	-0.14***
**Phosphorous**	0.21***	-0.19***	-0.21***
**Iron**	0.17***	-0.11***	-0.04
**Magnesium**	0.17***	-0.28***	-0.27***
**Zinc**	0.26***	-0.09**	-0.07*
**DDS**	0.34***		0.22***
**FVS**	0.43***		0.30***
**MAR**	0.32***		0.03
**Age 4–8 years**			
***Number (weighted n)***	***1510 (1405)***	***1510 (1405)***	***1510 (1405)***
**Carbohydrate (% E)**	-0.09**		0.02
**Protein (% E)**	-0.15***		-0.18***
**Fat (% E)**	0.17***		0.05
**Total carbohydrate**	0.40***		0.04
**Total protein**	0.24***		-0.21***
**Total fat**	0.39***		0.10**
**Total fibre**	0.06*		-0.28***
**Vitamin A**	0.08**	0.01	0.04
**Thiamin**	0.14***	-0.30***	-0.34***
**Riboflavin**	0.22***	0.07**	0.06*
**Niacin**	0.29***	0.04	0.03
**Vitamin B**_**12**_	0.04	0.00	0.01
**Vitamin B**_**6**_	0.30***	0.07**	0.05
**Folic acid**	0.22***	0.02	-0.01
**Pantothenic Acid**	0.24***	-0.05*	-0.08**
**Biotin**	0.09**	-0.11***	-0.08**
**Vitamin D**	0.16***	0.04	0.05
**Vitamin E**	0.12***	-0.04	-0.07*
**Vitamin C**	0.05*	0.03	-0.02
**Calcium**	0.20***	-0.04	0.00
**Phosphorous**	0.21***	-0.24***	-0.27***
**Iron**	0.13***	-0.13***	-0.12***
**Magnesium**	0.04	-0.41***	-0.45***
**Zinc**	0.22***	-0.11***	-0.14***
**DDS**	0.30***		0.14***
**FVS**	0.39***		0.21***
**MAR**	0.29***		-0.02

AS = Added sugar

†adjusted for kJ intake.

*Correlation/Partial correlation significant, p<0.05.

**Correlation/Partial correlation significant, p<0.01.

***Correlation/Partial correlation significant, p<0.0001.

In terms of mean daily micronutrient intakes (per 4.18MJ) by quartile of %EAS (Table 7) added sugar (%E) ranged from 1.0% of energy intake in Quartile 1 (Q1) to 19.2% of energy intake in Q4 for 1–3 year olds and 1.5% of energy intake in Q1 to 19.3% of energy intake in Q4 respectively for 4 to 8.9 year olds. Energy intakes did not differ significantly with increased %EAS in both age groups. The highest mean vitamin intakes per 4.18MJ were found in the lowest quartiles of %EAS intake for thiamin, pantothenic acid, biotin and vitamin E in 1–3 year olds (Table 7). In contrast riboflavin, niacin, vitamin B6 and folate intakes (per 4.18MJ) were significantly lower in the lowest quartile of %EAS intake. In the 4–8 year olds, higher vitamin intakes (per 4.18MJ) were found in the lowest quartile of added sugar (%EAS) intake only for thiamin. Niacin, folate and vitamin D intakes (per 4.18MJ) were found to be significantly lower in the lowest quartile of added sugar (%EAS) intake. Overall mean intakes for all minerals per 4.18MJ were significantly higher in the lowest quartile of sugar (%EAS) intakes for both age groups, with the exception of calcium for the 4–8 year olds.

**Table 7:** Mean daily micronutrient intakes per 4.18MJ of 1–3 year and 4–8 year olds according to quartiles of added sugar intake as a proportion of energy (% EAS).

	1–3 years					4–8 years				
Sugar quartile	Total	Q1	Q2	Q3	Q4	Total	Q1	Q2	Q3	Q4
***Number (weighted n)***	*1308 (795)*	*322 (199)*	*317 (199)*	*330 (199)*	*339 (198)*	*1510 (1405)*	*387 (351)*	*370 (352)*	*377 (351)*	*376 (351)*
**Energy (kJ) Mean**	4258.4	4162.2	4424.8	4306.7	4140.0	5494.6	5387.5	5651.7	5548.6	5390.6
**Energy (kJ) 95% CI** *	4128–4389	3866–4458	4219–4630	4085–4528	3884–4396	5345–5644	5084–5691	5435–5868	5290–5807	5061–5720
**AS (g) Mean**	21.8	2.9 [D]	14.1 [C]	25.2 [B]	45.2 [A]	30.4	5.8 [D]	20.0 [C]	33.6 [B]	62.1 [A]
**AS (g) 95% CI**	20.5–23.1	2.4–3.3	13.3–14.9	23.8–26.5	42.5–47.9	28.2–32.6	4.9–6.8	19.2–20.8	31.8–35.5	57.5–66.8
**AS (%E) Mean**	8.8	1.0 [D]	5.4 [C]	9.8 [B]	19.2 [A]	9.2	1.5 [D]	6.0 [C]	10.1 [B]	19.3 [A]
**AS (%E) 95% CI**	8.3–9.3	0.8–1.1	5.2–5.5	9.6–10.0	18.2–20.1	8.7–9.8	1.3–1.8	5.8–6.1	9.9–10.3	18.5–20.1
**Vitamin A (μgRE /4.18 MJ) Mean**	389.8	404.2	486.7	334.5	333.6	346.2	397.3 [A][B]	252.1 [B]	298.4 [A][B]	437.2 [A]
**Vitamin A (μgRE /4.18 MJ) 95% CI**	341.6–437.9	326–482.3	336.6–636.8	255.8–413.3	273.4–393.9	291.8–400.7	257.7–536.9	194.5–310	239.4–357	295.9–578.5
**Thiamin (mg /4.18 MJ) Mean**	0.59	0.64 [A]	0.62 [A]	0.58 [B]	0.51 [C]	0.57	0.66 [A]	0.59 [B]	0.55 [C]	0.50 [D]
**Thiamin (mg /4.18 MJ) 95% CI**	0.57–0.60	0.62–0.66	0.60–0.65	0.56–0.59	0.49–0.53	0.56–0.59	0.64–0.68	0.57–0.61	0.53–0.57	0.48–0.53
**Riboflavin (mg /4.18 MJ) Mean**	0.66	0.57 [B]	0.68 [A][B]	0.75 [A]	0.63 [A][B]	0.58	0.55	0.56	0.56	0.64
**Riboflavin (mg /4.18 MJ) 95% CI**	0.62–0.70	0.51–0.63	0.60–0.76	0.67–0.83	0.55–0.70	0.54–0.61	0.47–0.62	0.49–0.64	0.51–0.60	0.57–0.72
**Niacin (mg /4.18 MJ) Mean**	5.5	5.1 [B]	5.8 [A]	5.5 [A][B]	5.6 [A][B]	6.0	5.5 [B]	6.3 [A]	6.1 [A][B]	6.1 [A][B]
**Niacin (mg /4.18 MJ) 95% CI**	5.2–5.7	4.5–5.6	5.3–6.3	5.2–5.9	5.1–6.0	5.8–6.2	5.2–5.9	5.9–6.7	5.7–6.5	5.6–6.7
**Vitamin B12 (μg/4.18 MJ) Mean**	2.1	1.8	2.8	1.9	1.8	2.2	2.1	2.2	1.9	2.6
**Vitamin B12 (μg/4.18 MJ) 95% CI**	1.6–2.5	1.2–2.3	1.4–4.2	1.3–2.6	1.3–2.3	1.7–2.7	0.8–3.3	1.4–3.0	1.4–2.4	1.2–4.1
**Vitamin B6 (mg /4.18 MJ) Mean**	0.48	0.42 [B]	0.51 [A]	0.53 [A]	0.48 [A][B]	0.49	0.47	0.49	0.49	0.53
**Vitamin B6 (mg /4.18 MJ) 95% CI**	0.47–0.50	0.39–0.45	0.47–0.55	0.50–0.56	0.44–0.52	0.47–0.52	0.44–0.51	0.46–0.51	0.45–0.52	0.48–0.58
**Folate (μg /4.18 MJ) Mean**	93.9	80.4 [B]	99.8 [A]	101.5 [A]	93.9 [A][B]	115.0	101.2 [B]	124.7 [A]	114.8 [A][B]	119.5 [A]
**Folate (μg /4.18 MJ) 95% CI**	89.9–97.9	73.3–87.4	93.0–106.6	93.9–109.1	85.4–102.4	108.9–121.2	87.4–115.0	114.1–135	106.1–124	111.7–127.2
**Pantothenic (mg /4.18 MJ) Mean**	1.9	2.0 [A]	2.0 [A]	1.9 [A]	1.6 [B]	1.7	1.7	1.7	1.7	1.6
**Pantothenic (mg /4.18 MJ) 95% CI**	1.8–2.0	1.9–2.1	1.9–2.2	1.8–2.0	1.5–1.7	1.6–1.7	1.6–1.8	1.6–1.8	1.6–1.7	1.5–1.8
**Biotin (μg /4.18 MJ) Mean**	14.3	15.5 [A]	15.2 [A]	14.3 [A]	12.1 [B]	12.6	14.0	12.3	12.1	12.0
**Biotin (μg /4.18 MJ) 95% CI**	13.6–14.9	14.1–16.9	13.8–16.6	13.2–15.4	11.1–13.1	12.0–13.3	12.7–15.3	11.0–13.7	11.2–13.1	10.7–13.4
**Vitamin D (μg /4.18 MJ) Mean**	1.6	1.6	1.6	1.7	1.5	1.4	0.92 [B]	1.5 [A]	1.7 [A]	1.5 [A]
**Vitamin D (μg /4.18 MJ) 95% CI**	1.4–1.8	1.1–2.1	1.3–2.0	1.3–2.1	1.2–1.8	1.2–1.6	0.71–1.13	1.2–1.9	1.3–2.0	1.2–1.8
**Vitamin E (mg/ 4.18 MJ) Mean**	4.0	4.7 [A]	4.1 [A][B]	3.8 [B][C]	3.3 [C]	3.6	3.8	3.8	3.6	3.3
**Vitamin E (mg /4.18 MJ) 95% CI**	3.7–4.2	4.1–5.3	3.7–4.5	3.5–4.2	3.0–3.6	3.4–3.8	3.4–4.3	3.3–4.2	3.3–4.0	3.0–3.6
**Vitamin C (mg /4.18 MJ); 95% CI**	29.9	24.9	28.4	31.8	34.4	28.1	33.1	24.7	22.3	32.1
**Vitamin C (mg /4.18 MJ) 95% CI**	25.9–33.8	20.2–29.4	20.4–36.4	25.4–38.2	26.8–42.0	21.6–34.5	10.8–55.4	18.9–30.5	18.1–26.5	24.2–40.0
**Calcium (mg /4.18 MJ) Mean**	326.3	379.2 [A]	322.5 [B][C]	332.6 [A][B]	270.6 [C]	244.9	253.9	239.2	252.4	234.2
**Calcium (mg /4.18 MJ) 95% CI**	307.4–345.2	335.7–422.7	293.2–351.7	299.1–366.1	247.6–293.7	231.7–258.2	229.0–278.7	217.6–260.8	232.3–272.5	207.7–260.8
**Phosphorus(mg /4.18 MJ); 95% CI**	523.7–545.9	543.2–596.6	539.2–576.5	523.6–560.0	453.1–458.8	493.0–512.2	513.1–547.1	506.0–542.1	493.2–521.4	429.7–468.1
**Phosphorus (mg /4.18 MJ) Mean**	534.8	569.9 [A]	557.9 [A]	541.8 [A]	469.4 [B]	502.6	530.1 [A]	524.1 [A]	507.3 [A]	448.9 [B]
**Phosphorus (mg /4.18 MJ) 95% CI**	523.7–545.9	543.2–596.6	539.2–576.5	523.6–560.0	453.1–458.8	493.0–512.2	513.1–547.1	506.0–542.1	493.2–521.4	429.7–468.1
**Iron (mg /4.18 MJ) Mean**	5.0	5.5 [A]	5.2 [A][B]	4.7 [A][B]	4.6 [B]	5.2	6.1 [A]	5.3 [B]	5.0 [B]	4.6 [B]
**Iron (mg /4.18 MJ) 95% CI**	4.7–5.3	4.8–6.2	4.7–5.6	4.4–5.1	4.2–5.1	5.0–5.5	5.4–6.9	4.9–5.6	4.7–5.3	4.3–4.9
**Magnesium (mg /4.18 MJ) Mean**	161.9	175.9 [A]	169.4 [A][B]	159.2 [B]	143.1 [C]	160.8	183.2 [A]	168.9 [B]	159.3 [B]	131.6 [C]
**Magnesium (mg /4.18 MJ) 95% CI**	157.9–165.9	167.9–184	162.5–176.2	154.9–163.4	137.3–149.0	156.9–164.7	175.7–190.7	163.5–174	153.3–165	125.9–137.3
**Zinc (mg /4.18 MJ) Mean**	4.1	4.2 [A]	4.4 [A]	4.2 [A]	3.7 [B]	4.2	4.3 [A]	4.4 [A]	4.2 [A]	3.8 [B]
**Zinc (mg /4.18 MJ) 95% CI**	4.0–4.2	4.0–4.5	4.2–4.5	4.0–4.3	3.6–3.9	4.1–4.3	4.1–4.6	4.2–4.5	4.0–4.3	3.6–4.0

*95% CI = 95% Confidence interval

[A], [B], [C] AND [D]: significant difference between groups (quartiles of sugar, EAS % of energy intake) when letters are different

Bonferroni, p<0.05.

Table 8 shows that for children aged 1–3 years there was a significant trend for decreased stunting in the quartiles with higher sugar (%EAS) intakes [mean (95%CI) in terms of height-for-age Z score -1.22 (-1.42 - -1.01) in Q1 compared to -0.87 (-1.10–-0.64) in Q4 (p<0.05, Bonferroni multiple comparison test)]. The prevalence of stunting also showed a significant trend for decreased prevalence in the quartiles with higher sugar (%EAS) intakes [mean (95%CI) from 32.0 (27.4–36.6)% in Q1 compared to 24.8 (19.7–29.9)% in Q4 (Chi square p-value <0.05)]. The trend in mean height-for-age Z scores was also decreased in children aged 4–8 years, [mean (95% CI) height-for-age Z score -0.80 (-0.98 - -0.63) in Q1 compared to -0.58 (-0.78 - -0.37) in Q4 but not significantly so (Bonferroni p-value>0.05). However there was no trend seen in the mean prevalence of stunting with increasing sugar intakes (Table 9).

**Table 8:** The anthropometric status of children aged 1–3 years old nationally and by quartiles of added sugar intake as a percentage of energy (%EAS) (mean z-score and prevalence with two-sided confidence limits), according to WHO 2006/2007 sex specific z-scores, (male and female combined).

Sugar quartile	Total sample	Q1	Q2	Q3	Q4
***Number (weighted n)***	*1554(1097)*	*404*	*376 (272)*	*384 (275)*	*390 (269)*
**Height-for-age Z-score (mean)**	-1.11	-1.22 [B]	-1.29 [B]	-1.07 [AB]	-0.87 [A]
**Height-for-age Z-score (95% CI)**	-0.23	-0.41	-0.34	-0.32	-0.46
**Weight-for-age Z-score (mean),**	-0.32	-0.32 [AB]	-0.45 [B]	-0.22 [A]	-0.27 [AB]
**Weight-for-age Z-score (95% CI)**	-0.15	-0.25	-0.24	-0.25	-0.32
**BMI-for-age Z-score (mean)**	0.57	0.65	0.54	0.66	0.43
**BMI-for-age Z-score (95% CI)**	0.49 – 0.65	0.45 – 0.84	0.39 – 0.69	0.52 – 0.80	0.25 – 0.61
**Height-for-age Z-score <-2 (Stunting) %**	28	32	32.8	22.3	24.8
**Height-for-age Z-score <-2 (Stunting) 95% CI**	25.7 – 30.3	27.4 – 36.6	27.6 – 38.1	18.3 – 26.2	#19.7 – 29.9
**Weight-for-age Z-score <-2, %**	7.4	9.1	8.3	4.5	7.7
**Weight-for-age Z-score <-2, 95% CI**	6.1 – 8.8	6.2 – 11.9	5.6 – 11.0	2.6 – 6.5	4.9 – 10.6
**BMI-for-age Z-score >+2 to +3** * **%**	8.9	10	11.8	7.8	6
**BMI-for-age Z-score >+2 to +3** * **95% CI**	7.4 – 10.4	7.0–13.1	8.0 – 15.6	4.8 – 10.8	3.5 – 8.6
**BMI-for-age Z-score > +3** * **%**	4.8	6.8	3	5.1	4.2
**BMI-for-age Z-score > +3** * **% 95% CI**	3.6 – 5.9	4.0 – 9.5	1.4 – 4.6	2.9 – 7.3	2.2 – 6.2
**BMI-for-age Z-score >+2** * **(Overweight + obesity) %**	13.7	16.8	14.7	12.9	10.2
**BMI-for-age Z-score >+2** * **(Overweight + obesity) 95% CI**	11.6 – 15.7	12.2 – 21.4	10.7 – 18.8	9.1 – 16.6	6.8 – 13.6

* for children aged 1–5 years

[A], [B]: significant differences between groups (quartiles of sugar intake %EAS) when letters are different; Bonferroni, p<0.05.

#Significant relationship between different groups of added sugar (%EAS) intake and stunting, Chi square p<0.05.

**Table 9:** The anthropometric status of children aged 4–8 years old nationally and by quartiles of added sugar intake as a percentage of energy (%EAS) (mean z-score and prevalence with two-sided confidence limits), according to WHO 2006/2007 sex specific z-scores, (male and female combined).

Sugar quartile	Total sample	Q1	Q2	Q3	Q4
***Number (weighted n)***	*1045 (1103)*	*270 (280)*	*256 (280)*	*256 (272)*	*263 (272)*
**Height-for-age Z-score (mean)**	-0.71	-0.8	-0.83	-0.62	-0.58
**Height-for-age Z-score (95% CI)**	-0.26	-0.35	-0.47	-0.54	-0.41
**Weight-for-age Z-score (mean),**	-0.49	-0.66 [B]	-0.52 [AB]	-0.44 [AB]	-0.34 [A]
**Weight-for-age Z-score (95% CI)**	-0.2	-0.28	-0.38	-0.29	-0.42
**BMI-for-age Z-score (mean)**	-0.1	-0.25	-0.06	-0.09	0
**BMI-for-age Z-score (95% CI)**	-0.22 – 0.02	-0.36	-0.31 – 0.20	-0.29 – 0.11	-0.22 – 0.22
**Height-for-age Z-score <-2 (Stunting) %**	16	15.5	16.6	17.2	14.6
**Height-for-age Z-score <-2 (Stunting) 95% CI**	13.2 – 18.8	11.0 – 20.1	10.7 – 22.5	12.1 – 22.3	10.1 – 19.1
**Weight-for-age Z-score <-2, %**	8.1	10.4	7.1	7	8.1
**Weight-for-age Z-score <-2, 95% CI**	6.4 – 9.9	6.8 – 14.0	3.9 – 10.2	3.8 – 10.1	4.5 -11.7
**BMI-for-age Z-score >+1 to +2** * ; >+2 to +3** %	10.2	9.8	6.5	7.8	16.9
**BMI-for-age Z-score >+1 to +2** * ; >+2 to +3** 95% CI	8.3 – 12.2	5.9 – 13.7	3.6 – 9.4	4.6 – 11.0	11.7 – 22.1
**BMI-for-age Z-score >+2** * ; > +3** %	6.2	3.2	9.2	6.2	6.1
**BMI-for-age Z-score >+2** * ; > +3** 95% CI	3.8 – 8.5	0.9 – 5.5	3.0 – 15.4	3.0 – 9.3	3.1 – 9.1
**BMI-for-age Z-score >+1** * ; >+2** (Overweight + obesity) %	16.4	13	15.7	14	23.0&&
**BMI-for-age Z-score >+1** * ; >+2** (Overweight + obesity) 95% CI	13.3 – 19.5	8.7 – 17.3	9.1 – 22.2	9.7 – 18.3	16.2 – 29.8

* for children aged 5.1 years and older.

** for children aged 1–5 years.

[A], [B]: significant differences between groups (quartiles of sugar intake %EAS) when letters are different; Bonferroni, p<0.05.

&&Significant relationship between different groups of added sugar (%EAS) intake and (overweight + obesity), Chi square p<0.01.

There was a non-significant trend for a lower prevalence of overweight and obesity combined in 1–3 year old children (Table 8) in Q4 (highest %EAS) than in the other three quartiles (Q1-Q3), [mean (95%CI) % prevalence overweight 10.2 (6.8–13.6)% in Q4 compared to 16.8 (12.2–21.4)% in Q1]. However, the opposite was seen in the older children aged 4–8 years. The prevalence of overweight and obesity combined was significantly higher in the older children in Q4 (highest %EAS) than in the other three quartiles [mean (95%CI) % prevalence overweight 23.0 (16.2–29.8)% in Q4 compared to 13.0 (8.7–17.3)% in Q1, (p = 0.0063, Table 9)].

Decreased stunting and underweight was observed in both age groups in the urban areas compared with the rural areas, and for the 4–8 year old children the prevalence of overweight and obesity was higher in the urban than in the rural areas (S1 and S2 Tables). Children aged 1–3 years old in households with increased food expenditure showed decreased stunting and decreased underweight. For children aged 4–8 years old underweight was decreased and overweight and obesity was increased with increased household food expenditure (S1 and S2 Tables).

## Discussion

This analysis of food intakes, micronutrient intakes, micronutrient dilution and anthropometry in children consuming different amounts of added sugar in a developing country, extends previous findings published in 2003 [18], and affords a developing country’s more comprehensive perspective on previous studies conducted in children in the age range 1–8 years in the developed world [49–59].

The mean percentage contribution of added sugar to total energy (%EAS) was 9.1% in this study, which is just below the new and former WHO guidelines [2, 60] of a maximum of 10% energy from free sugars, and is also lower than that reported in national studies of children in developed countries (14.9 to 16.8%EAS) [51, 53, 57]. However, consumption was higher in urban children (10.3%EAS), and SES quartile analysis in both age groups showed children in the highest quartile had %EAS higher than the current WHO guidelines [2]. Free sugars include monosaccharides and disaccharides added to foods and beverages by the manufacturer, cook, or consumer, and sugars naturally present in honey, syrups, fruit juices and fruit concentrates as defined by WHO [2]. Thus free sugars as defined by FAO/WHO are similar to the definition for added sugars used in this paper, the main difference being that free sugars included sugars naturally present in fruit juice. Due to the limitations of the South African Food Composition Tables, which give no values for free sugars as per the WHO definition, we calculated free sugars intakes as using total carbohydrate in fruit juice and fruit concentrates (g). We found that the national intake of free sugars was 9.8% Energy from Free Sugars (% EFS) (29.8g/day); 11.4% EFS (36.2g/day) for urban children and 7.8% EFS (21.8g/day) for rural children. Urban children thus had intakes of free sugars above the 10% EFS WHO guidelines [2]; these guidelines also proposed a further reduction to 5% energy from free sugars as a conditional recommendation.

In this study the main source of added sugar for children was white sugar (60.1% of added sugar). In contrast, table sugar was not the main source of added sugar in the diets of children in developed countries, where a variety of processed foods and drinks,including soft drinks, carbonated beverages, fruit juice, fruit drinks, squash, yogurts/ cultured milks, chocolate, confectionary, high-fat desserts, cakes and sugary foods are the major sources of sugar [50–53, 55–58]. In 1999 in South Africa, the national contribution of added sugar intake from cool drinks (16.4%) was much lower than national studies in developed countries: 28% in the UK (4–18 year olds) [57]; and 35% in the USA (preschool children) [51]. A national study of school children in Norway found that soft drinks/ lemonade contributed 25–46% of added sugar in 4 year olds and 31–47% in 9 year olds (mean contributions in quartile 1 and quartile 4) [53]. However, in South Africa the percentage contribution to added sugar intakes from cool drinks (carbonated and squash types) in urban older children aged 7–8 years rose to 24.1%. Another difference from the data in developed countries [37–40] is that in this study in South Africa the increased sugar consumption is seen the higher SES households rather the lower SES households. This is similar to the data from Brazil [41]. The data in this study indicate a trend of increasing contribution of sugar intakes from cool drinks by age, urbanisation and improved SES and affords an important area of intervention in the current global efforts to reduce sugar consumption in line with WHO guidelines [2].

Concerns in relation to added sugar in the diet have been raised in the context of the potential displacement of nutrient dense foods from the diet [58]. The latter has been supported by data from developed countries where increased sugar intake in children has often been associated with a decreased intake of nutrient dense food groups; e.g. meat, fish eggs; grains, fruits and vegetables [55]; milk [49, 50]; fruits and vegetables at similar energy intakes [53]. However, the data in the current study in South African children is different. Increasing %EAS intakes were associated with increased dietary diversity scores, albeit the latter being in the low acceptable levels. Increased intakes of a number of nutrient rich food groups were found including meat, poultry and fish; eggs; as well as other fruits for both age groups and dairy in the older children. This may be due to confounding factors such as for instance increased income available to spend on food. Analysis of the money spent weekly on food showed a significant (chi square p <0.001) trend for both age groups for greater weekly expenditure on food in the households of the children consuming higher %EAS. Also households with increased food expenditure showed increased energy, protein and fat intakes in addition to increased added sugar intakes.

Associated with the higher intake of a number of food groups and combined with a lower intake of the cereals, roots and tubers group, there was no difference in energy intake observed with the increase in sugar (%EAS) intake. This is similar to other studies set in the developed world where energy intake did not rise significantly with higher sugar intakes [49, 52–54, 56, 58] although in three studies, there was a higher energy intake with a higher sugar intake in some groups [50, 56, 57]. The inconsistency of the findings in the available literature as well as in the present study on the role of sugar per se in increasing energy intake as opposed to an increase in total energy intake from all food items should be further investigated.

Another concern regarding higher sugar intakes has been the impact of high added sugar consumption on absolute micronutrient intakes and micronutrient dilution. Reviews of previous studies investigating the relationship between sugar and micronutrient intakes in developed countries has often been regarded as inconsistent [11, 14]. Methodological differences in studies (e.g. definition of sugar used, method of reporting sugar intakes and micronutrient intakes, the adjustment for differences in energy intakes [9] and differences in the selection of micronutrients investigated have added to the inconsistencies. To enable comparison of the findings of the present study with previous studies, we, in line with most of the studies in children in developed countries, used quartiles of % energy from added sugar for most of the analyses (S3 and S4 Tables), and reported absolute micronutrient intakes and micronutrient density as micronutrient intakes per 4.184 MJ intake to allow for differences in energy intakes. However, we would argue that it is not to be expected that the impact of sugar intake on micronutrient intakes and micronutrient dilution would be uniform, because the quality of the baseline diet and food patterns concurrent with changes in sugar intakes would be expected to lead to differing effects. Rennie and Livingstone [9] for instance have stated that “The risk of low micronutrient intakes appears to be greatest in diets that are characterized by high % energy from sugars at low levels of energy intake.” Therefore, differential effects could be expected for children, adults and the elderly. However, the reviews generally have considered both studies in adults and in children together thus highlighting the need to consider the studies in children separately.

Our study and other studies in children of similar ages (summarised in S3 and S4 Tables), show, that for most micronutrients, intakes (either absolute or per unit energy intake) are reduced when sugar intakes are high [49–59]. The previous studies showed overall consistent decreases in intakes of several B vitamins, and for the minerals magnesium, zinc and phosphorous. Vitamins A, E, folate, and calcium and iron intakes were decreased with increased sugar intakes in most studies. Exceptions were found for vitamins C and D where the effect was much less consistent [49–59].

In the current study all the minerals showed micronutrient dilution with increase in sugar intake in both age groups, except for calcium in the 4–8 year old children. Thiamin, pantothenic acid, biotin and vitamin E showed micronutrient dilution in both age groups. The micronutrients considered to be “diluted” in the diet, were also found to be affected in other studies in children and adolescents in the developed world (S3 and S4 Tables) [49–59]. In the present study, absolute micronutrient intakes rose with increasing absolute added sugar consumption for all micronutrients except vitamin B_12_ in both age groups and magnesium in the 4–8 year old children. This is consistent with the findings of increased energy intake, increased dietary diversity and increased consumption of a variety of food groups with an increased intake of added sugar. The increase in energy intake seen over the four quartiles of absolute added sugar consumption in our study, was a 1885–2505 kJ/day increase, of which 931–1116 kJ/day was attributable to the added sugar (for the 1–3 and 4–8 year olds respectively). Even though the absolute micronutrient intakes rose with absolute sugar intake in the present study, micronutrient intakes of children were low at all levels of added sugar in the quartiles as shown by a mean MAR of 64.7 and 62.5 (children aged 1–3 years and 4–8 years respectively).

The findings of the present study are also similar to those of the regional THUSA study in South African adults which found a small but significant nutrient dilution effect for a number of micronutrients in men and women with higher %EAS intakes [17]. Another regional South African study in the elderly found micronutrient dilution in women with higher %EAS, but not in men [16]. The clinical significance of the micronutrient dilution concept, if any, in South Africa, should be seen against the mandatory multiple micronutrient food fortification policy enacted in 2003. Micronutrient fortification of staple foods, a tried and tested long-term efficacious policy in addressing micronutrient deficiencies, has recently been shown to be associated with significant improvements in micronutrient status, at least in folate status, as well as vitamin A and iron status in both children and women of reproductive age [25, 27].

We also addressed the issue of stunting as well as overweight and obesity in South African children in relation to %EAS in the diet. The decreased stunting prevalence in the children in the quartiles with the higher %EAS (statistically significant in the 1–3 year old children only), may indeed be related to the increased dietary diversity. However, it is of concern that in the 4–8 year old children the prevalence of overweight and obesity was significantly higher in the highest %EAS quartile than in the other three quartiles, consistent with previous analysis of the children aged 6–9 years in this same dataset [18]. Studies in children in the developed countries have shown contradictory results. In Finland, among children of similar age groups, Ruottinen *et al*. [49] found that BMI was higher in the high sugar consumers aged 1–3 years of age and lower in the high sugar consumers aged 5–9 years of age. Also in Norway, Overby *et al*. [53] found that BMI in boys aged 4 years was higher with increased %EAS, whereas it was lower with increased %EAS in 13 year old girls. With regard to South Africans, a cohort study in adults showed that in the period 2005 to 2010 the consumption of added sugar and sugar sweetened beverages increased dramatically, with an increased BMI in higher sugar consumers, presumably partly due to increased total energy intakes [61]. Data from prospective studies in the literature showed sugar sweetened beverage consumption to be the most consistent dietary factor in association with subsequent increases in adiposity [31]. Also randomized controlled intervention trials in children lasting 6 months to 2 years have shown that lowering the intake of soft drinks reduced weight gain [28]. In the South African context at least a focus on interventions preventing further increases in the consumption of sugar, derived from added sugar or sweetened beverages consumption would appear to be appropriate for the reduction of the prevalence of overweight and obesity in children.

The main limitation of our secondary analysis is that the latter was based on a cross sectional observational national survey and thus does not allow any conclusions about causal relationships. The 24 hour dietary recalls were completed by interviewing the caregivers of the children. This may have led to under-reporting, particularly of snacks and sugar sweetend beverages consumed away from the home environment. Although this study was carried out in 1999, in light of the increasing concerns about sugar in the diet globally and, in the national context, the recent national data from the SANHANES 1 [27] documenting that less than half (42.1%) of the population had a low sugar consumption score, with 27.0% of the youngest segment of the population (15–24 years of age) having a high sugar consumption score), the authors felt that the secondary analysis of older national data would afford additional baseline perspective that would contribute to the current debate on appropriate policy intervention strategies to address the need to decrease the intake of sugar in the country. Also it is the first ever national dietary survey of children in South Africa and only national dietary survey which allows calculation of micronutrient intakes. Dietary diversity remains a concern [27] and although the status of some micronutrients is improving in high risk groups [27] micronutrient intakes in general remain of concern in South Africa in the context of low dietary variety, poor dietary practices and choices and inadequate intakes of energy [26, 27]. Monitoring of added sugar intakes in children in South Africa in relation to micronutrient intakes and status and overweight/obesity are important areas of future research. When considering these dietary issues in conjunction with the current dual nutritional burden of stunting and overweight/obesity in South African children [27], the findings of this study suggest that there is a need for the diet of children to be considered in terms broader than current policies as a whole allow. Micronutrient intakes should increase, e.g through food fortification, and nutrition education [62]. Measures in line with other countries in transition [63] should emphasise the consumption of micronutrient rich foods and also actively promote the reduction in sugar intake. Suggestions for reducing sugar consumption include the prohibition of advertising of foods and beverages high in sugar and restriction of the use of advertising techniques which appeal specifically to children during children and family television viewing times [64].

## Conclusion

DDS and FVS (but not MAR), energy intakes and the absolute intakes of micronutrients were positively partially correlated with added sugar intakes (adjusted for energy intake). Overall protein, fibre and several micronutrient intakes adjusted for energy intake were negatively associated with percentage of energy from added sugar intakes showing micronutrient dilution. Overweight and obesity was increased with higher added sugar intakes in the 4–8 year old children.

## Supporting Information

S1 Table The anthropometric status of children aged 1–3 years nationally, by geotype and by categories of money spent by household on food weekly (SES) (mean z-score and prevalence with two-sided confidence limits), according to WHO 2006/2007 sex specific z-scores, (male and female combined) (DOCX)

S2 Table The anthropometric status of children aged 4–8 years old nationally, by geotype and by categories of money spent by household on food weekly (SES) (mean z-score and prevalence with two-sided confidence limits), according to WHO 2006/2007 sex specific z-scores, (male and female combined) (DOCX)

S3 Table Summary of relationships between higher sugar intakes and vitamin intakes in children. (DOCX)

S4 Table Summary of relationships between higher sugar intakes and mineral intakes in children. (DOCX)
