# Supplementary material for: Added Sugar, Macro- and Micronutrient Intakes and Anthropometry of Children in a Developing World Context
Source: PLoS One. 2015 Nov 11;10(11):e0142059. doi: 10.1371/journal.pone.0142059 (PMC4641690; doi:10.1371/journal.pone.0142059)
Supplement: S1 Table — (DOCX) [file pone.0142059.s001.docx]

**S1 Table.** The anthropometric status of children aged 1-3 years nationally, by geotype and by categories of money spent by household on food weekly (SES) (mean z-score and prevalence with two-sided confidence limits), according to WHO 2006/2007 sex specific z-scores, (male and female combined)

| **1-3 yrs** |  | **Total sample** | **SA Urban** | **SA Rural** | **SES 1** | **SES 2** | **SES 3** | **SES 4** |
| --- | --- | --- | --- | --- | --- | --- | --- | --- |
|  | ***Number (weighted n)* ^@^** | 1554 (1097) | 781 (605) | 773 (492) | 426 (305) | 301 (205) | 301 (216) | 302 (228) |
|  | **Height-for-age Z-score (mean)** | -1.11 | -0.93^$$$^ | -1.33 | -1.24 [B] | -1.22 [B] | -1.06 [B] | -0.61 [A] |
|  | **Height-for-age Z-score (95% CI)** | -1.23 - -1.00 | -1.09 - -0.78 | -1.49 - -1.17 | -1.42 - -1.06 | -1.47 - -0.97 | -1.27 - -0.85 | -0.85 - -0.38 |
|  | **Weight-for-age Z-score (mean),** | -0.32 | -0.18^$$$^ | -0.48 | -0.42 [B] | -0.37 [B] | -0.23 [A][B] | -0.03 [A] |
|  | **Weight-for-age Z-score (95% CI)** | -0.39 - -0.24 | -0.30 - -0.06 | -0.57 - -0.40 | -0.54 - -0.30 | -0.55 - -0.19 | -0.39 - -0.08 | -0.21 – 0.15 |
|  | **BMI-for-age Z-score (mean)** | 0.57 | 0.60 | 0.54 | 0.55 | 0.59 | 0.64 | 0.52 |
|  | **BMI-for-age Z-score (95% CI)** | 0.49 – 0.65 | 0.48 – 0.71 | 0.42 – 0.66 | 0.42 – 0.68 | 0.37 – 0.81 | 0.47 – 0.80 | 0.34 – 0.71 |
|  | **Height-for-age Z-score <-2 (Stunting) %** | 28.0 | 22.8^###^ | 34.5 | 32.4^&&^ | 30.7 | 26.4 | 16.1 |
|  | **Height-for-age Z-score <-2 (Stunting) 95% CI** | 25.7 – 30.3 | 19.9 – 25.6 | 30.7 – 38.3 | 27.5 – 37.3 | 24.6 – 36.8 | 21.3 – 31.5 | 11.5 – 20.8 |
|  | **Weight-for-age Z-score <-2, %** | 7.4 | 5.8^#^ | 9.5 | 7.2 | 9.2 | 6.5 | 2.9 |
|  | **Weight-for-age Z-score <-2 95% CI** | 6.1 – 8.8 | 4.0 – 7.5 | 7.3 – 11.6 | 4.7 – 9.7 | 5.4 – 13.1 | 3.6 – 9.4 | 1.0 – 4.8 |
|  | **BMI-for-age Z-score >+2 to +3*%** | 8.9 | 8.7 | 9.2 | 9.3 | 9.3 | 9.5 | 7.3 |
|  | **BMI-for-age Z-score >+2 to +3* 95% CI** | 7.4 – 10.4 | 6.6 – 10.8 | 7.0 – 11.4 | 6.5 – 12.0 | 6.0 – 12.5 | 5.6 – 13.5 | 3.9 – 10.7 |
|  | **BMI-for-age Z-score > +3* %** | 4.8 | 4.7 | 4.8 | 3.9 | 7.7 | 4.8 | 5.0 |
|  | **BMI-for-age Z-score > +3* % 95% CI** | 3.6 - 5.9 | 3.1 – 6.3 | 3.0 - 6.6 | 1.9 – 6.0 | 4.4 – 10.9 | 2.1 – 7.6 | 2.5 – 7.5 |
|  | **BMI-for-age Z-score >+2* (Overweight + obesity) %** | 13.7 | 13.4 | 14.0 | 13.2 | 17.0 | 14.4 | 12.4 |
|  | **BMI-for-age Z-score >+2* (Overweight + obesity) 95% CI** | 11.6 – 15.7 | 10.5 – 16.3 | 11.1 – 17.0 | 9.9 – 16.5 | 12.7 – 21.2 | 10.3 – 18.5 | 7.8 – 16.9 |

*for children aged 1-5 years

[A], [B]: significant differences between SES groups when letters are different; Bonferroni, p<0.05

^#^Significant relationship between urban and rural groups and underweight, Chi square p<0.05

^###^Significant relationship between urban and rural groups and stunting, Chi square p<0.0001

^&&^Significant relationship between different SES groups and stunting, Chi square p<0.01

^$$$^Significant difference between urban and rural groups, independent t-test , p<0.0001

* 95% CI = 95% Confidence Intervals: LCI=Lower confidence interval; UCI=Upper confidence interval

[A], [B],[C],[D]: Significant differences between SES groups when letters are different; Bonferroni, p<0.05

@ Fewer subjects reported money spent on food.
