# Supplementary material for: Added Sugar, Macro- and Micronutrient Intakes and Anthropometry of Children in a Developing World Context
Source: PLoS One. 2015 Nov 11;10(11):e0142059. doi: 10.1371/journal.pone.0142059 (PMC4641690; doi:10.1371/journal.pone.0142059)
Supplement: S2 Table — (DOCX) [file pone.0142059.s002.docx]

**S2 Table.** The anthropometric status of children aged 4-8 years old nationally, by geotype and by categories of money spent by household on food weekly (SES) (mean z-score and prevalence with two-sided confidence limits), according to WHO 2006/2007 sex specific z-scores, (male and female combined)

| **4-8 yrs** |  | **Total sample** | **SA Urban** | **SA Rural** | **SES 1** | **SES 2** | **SES 3** | **SES 4** |
| --- | --- | --- | --- | --- | --- | --- | --- | --- |
|  | ***Number (weighted n)* ^@^** | 1045 (1103) | 511 (603) | 534 (500) | 229 (240) | 224 (242) | 251 (260) | 210 (226) |
|  | **Height-for-age Z-score (mean)** | -0.71 | -0.62^$^ | -0.82 | -0.69 [A][B] | -0.95 [B] | -0.74 [A][B] | -0.35 [A] |
|  | **Height-for-age Z-score (95% CI)** | -0.84 - -0.58 | -0.82 - -0.42 | -0.99 - -0.64 | -0.98 - -0.39 | -1.24 - -0.66 | -0.94 - -0.54 | -0.61 - -0.08 |
|  | **Weight-for-age Z-score (mean),** | -0.49 | -0.32^$$$^ | -0.69 | -0.68 [B] | -0.65 [B] | -0.44 [B] | -0.02 [A] |
|  | **Weight-for-age Z-score (95% CI)** | -0.59 - -0.39 | -0.48 - -0.16 | -0.80 - -0.59 | -0.84 - -0.51 | -0.82 - -0.48 | -0.63 - -0.24 | -0.25 – 0.22 |
|  | **BMI-for-age Z-score (mean)** | -0.10 | 0.06^$$^ | -0.29 | -0.39 [C] | -0.12 [B][C] | 0.00 [A][B] | 0.26 [A] |
|  | **BMI-for-age Z-score (95% CI)** | -0.22 - 0.02 | -0.14 – 0.24 | -0.42 - -0.16 | -0.63 - -0.14 | -0.43 – 0.19 | -0.24 – 0.25 | 0.04 – 0.48 |
|  | **Height-for-age Z-score <-2 (Stunting) %** | 16.0 | 12.4^##^ | 20.4 | 19.5 | 18.0 | 17.0 | 10.6 |
|  | **Height-for-age Z-score <-2 (Stunting) 95% CI** | 13.2 – 18.8 | 8.3 – 16.5 | 16.5 – 24.3 | 13.6 – 25.4 | 10.4 – 25.6 | 11.6 – 22.4 | 6.6 – 14.7 |
|  | **Weight-for-age Z-score <-2, %** | 8.1 | 6.2^#^ | 10.4 | 9.1^&^ | 10.2 | 5.5 | 4.1 |
|  | **Weight-for-age Z-score <-2, 95% CI** | 6.4 – 9.9 | 4.1 – 8.4 | 7.5 – 13.3 | 4.9 – 13.2 | 6.2 – 14.2 | 2.6 – 8.3 | 1.1 – 7.1 |
|  | **BMI-for-age Z-score >+1 to +2*; >+2 to +3** %** | 10.2 | 10.5 | 9.8 | 6.2 | 10.9 | 11.4 | 14.4 |
|  | **BMI-for-age Z-score >+1 to +2*; >+2 to +3** 95% CI** | 8.3 – 12.2 | 7.6 – 13.5 | 7.4 – 12.3 | 2.7 – 9.8 | 6.1 – 15.7 | 7.4 – 15.5 | 9.0 – 19.7 |
|  | **BMI-for-age Z-score >+2*; > +3** %** | 6.2 | 8.2 | 3.7 | 4.5 | 6.7 | 5.7 | 9.4 |
|  | **BMI-for-age Z-score >+2*; > +3** 95% CI** | 3.8 – 8.5 | 4.0 – 12.4 | 1.9 – 5.4 | 1.2 – 7.8 | 0.8 – 12.6 | 0.3 – 11.2 | 4.5 – 14.3 |
|  | **BMI-for-age Z-score >+1 *; >+2** (Overweight + obesity) %** | 16.4 | 18.8^#^ | 13.5 | 10.7^&&^ | 17.6 | 17.2 | 23.8 |
|  | **BMI-for-age Z-score >+1 *; >+2** (Overweight + obesity) 95% CI** | 13.3 – 19.5 | 13.6 – 23.9 | 10.5 – 16.6 | 5.1 – 16.4 | 10.0 – 25.1 | 11.0 – 23.4 | 17.1 – 30.5 |

* for children aged 5.1 years and older

** for children aged 1-5 years

[A], [B]: significant differences between SES groups when letters are different; Bonferroni, p<0.05

^$^Significant difference between urban and rural groups, independent t-test , p<0.05

^$$^Significant difference between urban and rural groups, independent t-test , p<0.01

^$$$^Significant difference between urban and rural groups, independent t-test , p<0.0001

^#^Significant relationship between urban and rural groups, Chi square p<0.05

^##^Significant relationship between urban and rural groups and stunting, Chi square p<0.01

^&^Significant relationship between different SES groups and underweight, Chi square p<0.05

^&&^Significant relationship between different SES groups and (overweight + obesity), Chi square p<0.01

* 95% CI = 95% Confidence Intervals: LCI=Lower confidence interval; UCI=Upper confidence interval

[A], [B],[C],[D]: Significant differences between SES groups when letters are different; Bonferroni, p<0.05

@ Fewer subjects reported money spent on food.
