# Supplementary material for: Added Sugar, Macro- and Micronutrient Intakes and Anthropometry of Children in a Developing World Context
Source: PLoS One. 2015 Nov 11;10(11):e0142059. doi: 10.1371/journal.pone.0142059 (PMC4641690; doi:10.1371/journal.pone.0142059)
Supplement: S3 Table — (DOCX) [file pone.0142059.s003.docx]

**S3 Table**: Summary of relationships` between higher sugar intakes and vitamin intakes in children.

|  |  | **VITAMINS** | | | | | | | | | | | |
| --- | --- | --- | --- | --- | --- | --- | --- | --- | --- | --- | --- | --- | --- |
| **Study Reference Number, Method of Sugar Analysis, Age of children** | **Method of micro-nutrient analysis** | **Bold** **indicates** **significant decrease**, normal typeface no significant change, *italics significant increase*, no entry means that the vitamin was not studied. | | | | | | | | | | | |
| [49] Ruottinen et al; 3 groups %ES, 13 month to 9 year olds | Absolute intakes |  |  |  | **B3** |  |  |  |  |  | C | D | **E** |
| [50] Gibson; %ENMES quintiles, 1.5 -4.5 year olds | Absolute intake |  | **B1** | **B2** | **B3** |  |  | Folate |  |  | *C* | D |  |
| [51] Kranz et al; 5 groups %EAS, 2-3 & 4-5 year olds | Absolute intakes | **A** |  |  |  | **B6** | **B12** | **Folate** |  |  | **C** |  |  |
| [52] Erkkola et al; %EAS quartiles, 3 year olds | Absolute intakes | A | **B1** | **B2** | **B3** | B6 | **B12** | **Folate** |  |  | C | **D** | **E** |
| [53] Overby et al; %EAS quartiles, 4 year olds | Absolute intakes | **A** | **B1** | **B2** |  |  |  |  |  |  | C | **D** | E |
| [53] Overby et al; %EAS quartiles, 9 year olds | Absolute intakes | **A** | **B1** | **B2** |  |  |  |  |  |  | C | **D** | **E** |
| [54] Farris et al; TSg/1000 kcal quartiles, 10 year olds | Absolute intakes |  |  |  | **B3** | **B6** |  |  |  |  | C | *D* | **E** |
| [55] Alexy et al; %EAS quintiles, 2-18 year olds | Absolute intakes as % Reference Values | **A** | **B1** |  |  |  |  | **Folate** |  |  | **C** |  |  |
| [56] Lyhne and Ovesen; %EAS quintiles, 4- 14 year olds | Intakes /1000kcal (micronutrient density) | **A** | **B1** | **B2** | **B3** | **B6** | **B12** | **Folate** |  |  | C | **D** | **E** |
| [57] Gibson and Boyd, %EAS quintiles, 4-18 year olds | Absolute intakes as % RNI | **A** | **B1** | **B2** | **B3** | B6 | **B12** | **Folate** |  |  | C |  | *E* |
| [58] Joyce and Gibney; %EAS Tertiles, 5-12 year olds | Intakes / 10 MJ (micronutrient density) | **A** | **B1** | **B2** | **B3** | **B6** | **B12** | **Folate** | **Pantothenic Acid** | **Biotin** | **C** | D | **E** |
| [59] Forshee and Storey 2001, modelling of data 6-11 year olds | Absolute intakes as %RDA | A |  |  |  |  |  | *Folate* |  |  | *C* |  |  |
| [53] Overby et al; %EAS quartiles, 13 year olds | Absolute intakes | **A** | **B1** | **B2** |  |  |  |  |  |  | **C** | **D** | **E** |
| ***This study***; %EAS quartiles, 1-3 year olds | Intakes / 4.18 MJ (micronutrient density) | A | **B1** | *B2* | *B3* | *B6* | B12 | *Folic Acid* | **Pantothenic Acid** | **Biotin** | C | D | **E** |
| ***This study***; %EAS quartiles , 4-8 year olds | Intakes/ 4.18MJ (micronutrient density) | A | **B1** | B2 | *B3* | B*6* | B12 | *Folic Acid* | Pantothenic Acid | Biotin | C | *D* | E |
| ***This study***; correlation, Absolute added sugar (g), 1-3 year olds | Absolute intakes | *A* | *B1* | *B2* | *B3* | *B6* | B12 | *Folic Acid* | *Pantothenic Acid* | *Biotin* | *C* | *D* | *E* |
| ***This study***; correlation, Absolute added sugar (g), 4-8 year olds | Absolute intakes | *A* | *B1* | *B2* | *B3* | *B6* | B12 | *Folic Acid* | *Pantothenic Acid* | *Biotin* | *C* | *D* | *E* |
| ***This study***; partial correlation, Absolute added sugar (g), 1-3 year olds | Intakes adjusted for kJ intake | A | **B1** | *B2* | *B3* | *B6* | B12 | Folic Acic | **Pantothenic Acid** | **Biotin** | *C* | D | **E** |
| ***This study***; partial correlation, Absolute added sugar (g), 4-8 year olds | Intakes adjusted for kJ intake | A | **B1** | *B2* | B3 | B6 | B12 | Folic Acid | **Pantothenic Acid** | **Biotin** | C | D | **E** |

%ES = % energy intake from sugar; %ENMES = % energy intake from non-milk extrinsic sugars %EAS = % energy intake from added sugars; TS = Total sugars;

Study Reference numbers relate to references listed in article “Added sugar, macro- and micronutrient intakes and anthropometry of children in a developing world context”
