# Supplementary material for: Added Sugar, Macro- and Micronutrient Intakes and Anthropometry of Children in a Developing World Context
Source: PLoS One. 2015 Nov 11;10(11):e0142059. doi: 10.1371/journal.pone.0142059 (PMC4641690; doi:10.1371/journal.pone.0142059)
Supplement: S4 Table — (DOCX) [file pone.0142059.s004.docx]

**S4 Table**: Summary of relationships between higher sugar intakes and mineral intakes in children.

|  |  | **MINERALS** | | | | | | | | | | | |
| --- | --- | --- | --- | --- | --- | --- | --- | --- | --- | --- | --- | --- | --- |
| **Study Reference Number, Method of Sugar Analysis, Age of children** | **Method of micro-nutrient analysis** | **Bold** indicates significant decrease/negative correlation, normal typeface no significant change/correlation, *italics* significant increase/positive correlation. No entry: the mineral was not studied | | | | | | | | | | | |
| [49] Ruottinen et al; 3 groups %ES, 13 month to 9 year olds | Absolute intakes | **Ca** | **Fe** |  | **Zn** |  |  |  |  |  |  |  |  |
| [50] Gibson; %ENMES quintiles, 1.5 -4.5 year olds | Absolute intake | **Ca** | Fe |  | **Zn** |  |  |  |  |  |  |  |  |
| [51] Kranz et al; 5 groups %EAS, 2-3 & 4-5 year olds | Absolute intakes | **Ca** | **Fe** | **Mg** | **Zn** |  | **Na** |  |  |  |  |  |  |
| [52] Erkkola et al; %EAS quartiles, 3 year olds | Absolute intakes | **Ca** | **Fe** | **Mg** | **Zn** | **P** |  | **K** |  | **I** | **Se** | Cu |  |
| [53] Overby et al; %EAS quartiles, 4 year olds | Absolute intakes | **Ca** | **Fe** |  |  |  |  |  |  |  |  |  |  |
| [53] Overby et al; %EAS quartiles, 9 year olds | Absolute intakes | **Ca** | **Fe** |  |  |  |  |  |  |  |  |  |  |
| [54] Farris et al; TSg/1000 kcal quartiles, 10 year olds | Absolute intakes | *Ca* | **Fe** |  | **Zn** |  |  |  |  |  |  |  |  |
| [55] Alexy et al; %EAS quintiles, 2-18 year olds | Absolute intakes as % Reference Values | **Ca** | **Fe** |  |  |  |  |  |  |  |  |  |  |
| [56] Lyhne and Ovesen; %EAS quintiles, 4- 14 year olds | Intakes /1000kcal (micronutrient density) | **Ca** | **Fe** | **Mg** | **Zn** | **P** | **Na** | **K** |  | **I** | **Se** |  |  |
| [57] Gibson and Boyd, %EAS quintiles, 4-18 year olds | Absolute intakes as % RNI | **Ca** | **Fe** | **Mg** | **Zn** |  |  |  |  |  |  |  |  |
| [58] Joyce and Gibney; %EAS Tertiles, 5-12 year olds | Intakes / 10 MJ (micronutrient density) | **Ca** | **Fe** | **Mg** | **Zn** | **P** | **Na** | **K** | **Cl** | **I** | **Se** | **Cu** | **Mn** |
| [59] Forshee and Storey 2001, modelling of data 6-11 year olds | Absolute intakes as %RDA | Ca | *Fe* |  |  |  |  |  |  |  |  |  |  |
| [53] Overby et al; %EAS quartiles, 13 year olds | Absolute intakes | **Ca** | **Fe** |  |  |  |  |  |  |  |  |  |  |
| ***This study***; %EAS quartiles, 1-3 year olds | Intakes / 4.18 MJ (micronutrient density) | **Ca** | **Fe** | **Mg** | **Zn** | **P** |  |  |  |  |  |  |  |
| ***This study***; %EAS quartiles , 4-8 year olds | Intakes/ 4.18MJ (micronutrient density) | Ca | **Fe** | **Mg** | **Zn** | **P** |  |  |  |  |  |  |  |
| ***This study***; correlation, Absolute added sugar (g), 1-3 year olds | Absolute intakes | *Ca* | *Fe* | *Mg* | *Zn* | *P* |  |  |  |  |  |  |  |
| ***This study***; correlation, Absolute added sugar (g), 4-8 year olds | Absolute intakes | *Ca* | *Fe* | Mg | *Zn* | *P* |  |  |  |  |  |  |  |
| ***This study***; partial correlation, Absolute added sugar (g), 1-3 year olds | Intakes adjusted for kJ intake | **Ca** | Fe | **Mg** | **Zn** | **P** |  |  |  |  |  |  |  |
| ***This study***; partial correlation, Absolute added sugar (g), 4-8 year olds | Intakes adjusted for kJ intake | Ca | **Fe** | **Mg** | **Zn** | **P** |  |  |  |  |  |  |  |

%ES = % energy intake from sugar; %ENMES = % energy intake from non-milk extrinsic sugars %EAS = % energy intake from added sugars; TS = Total sugars;

Study Reference numbers relate to references listed in article “Added sugar, macro- and micronutrient intakes and anthropometry of children in a developing world context”
